# Supplementary material for: A whole-animal phenotypic drug screen identifies suppressors of atherogenic lipoproteins
Source: eLife. 2026 Aug 27;14:RP105314. doi: 10.7554/eLife.105314 (PMC13521566; doi:10.7554/eLife.105314)
Supplement: Supplementary file 2 [file elife-105314-code1.zip › JHDL_primary_result/Myristic acid/Myristic acid_JHDL_27_034_054_2019_11_08_12_13.pdf]

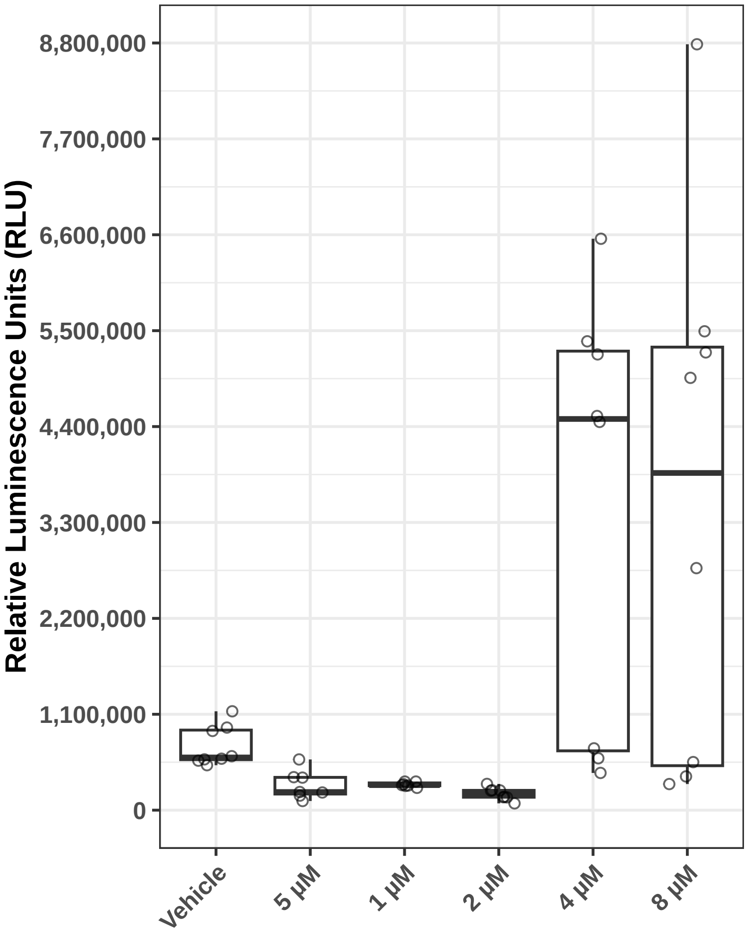

Relative Luminescence Units (RLU)
8,800,000
7,700,000
6,600,000
5,500,000
4,400,000
3,300,000
2,200,000
1,100,000
0
Vehicle
5 µM
1 µM
2 µM
4 µM
8 µM
